# Allelopathic effects of four spontaneous plants from arid regions on the germination and growth of selected weeds and crops

**DOI:** 10.3389/fphar.2026.1746401

**Published:** 2026-05-20

**Authors:** Malak Harabi, Walid Elfalleh, Dhawya Mergby, Abdelkarim Ben Arfa, Ilyes Saidi, Rania Kaddachi, Hamdi Bendif, Sulaiman A. Alsalamah, Samira Missaoui, Hichem Ben Jannet, Hanen Najjaa

**Affiliations:** 1 Laboratory of Pastoral Ecosystems and Valorization of Spontaneous Plants and Microorganisms, Institute of Arid Regions, Medenine, Tunisia; 2 Department of Biology, College of Science, Imam Mohammad Ibn Saud Islamic University (IMSIU), Riyadh, Saudi Arabia; 3 Laboratory of Heterocyclic Chemistry, Natural Products and Reactivity, Faculty of Science of Monastir, University of Monastir, Monastir, Tunisia; 4 Higher School of Health Sciences and Techniques of Sfax (ESSTSS), University of Sfax, Sfax, Tunisia; 5 Laboratory of Extremophile Plants, Centre of Biotechnology of Borj Cedria, Hammam-Lif, Tunisia

**Keywords:** allelopathy, antioxidant capacity, arid-zone plants, germination test, growth test, LC-MS analysis, molecular docking, phenolic compounds

## Abstract

The study investigates the allelopathic potential of the aqueous extracts of four plants species; *Cleome arabica* L., *Diplotaxis harra* (Forssk.) Boiss., *Nerium oleander* L., and *Retama raetam* (Forssk.) Webb. Collected from Tunisian arid regions. The effects of these extracts were evaluated on seed germination and seedling growth of two weeds (*Setaria verticillata* L. and *Sisymbrium irio* L.) and two crops (*Solanum lycopersicum* L. and *Capsicum annuum* L.) at different concentrations. Aqueous extracts, prepared by cold maceration, were analyzed for their allelochemicals content, mainly polyphenols, flavonoids, and tannins and further characterized by LC-MS analysis. Antioxidant capacity was assessed using total antioxidant and antiradical capacities. *Retama raetam,* followed by *N. oleander*, exhibited the highest phenolic compounds content as well as the strongest total antioxidant capacity. Both species strongly inhibited the germination of *S. verticillata* and *S. irio,* particularly at 20 g/L. In contrast, crop species showed greater tolerance, with *S. lycopersicum* maintaining germination rates above 50%, even at the highest concentrations. Growth bioassays revealed that roots were more sensitive than shoots, especially in weed species, indicating both organ selectivity and dose-dependent responses. Furthermore, allelopathic activity was supported by an *in silico* molecular docking study. The main metabolites of the studied samples were docked into the active pocket of glutathione S-transferase (GST; PDB: 1BX9) and 4-hydroxyphenylpyruvate dioxygenase (hydroxyphenylpyruvate dioxygenase; PDB: 6J63). These findings highlight the potential of arid-land allelopathic plants as natural herbicides, with *R. raetam* and *N. oleander* emerging as promising candidates for sustainable weed management.

## 1 Introduction

Weeds are undesirable herbaceous plants with no recognized economic or ecological value. They disrupt agroecosystems by interfering with agricultural operations, increasing cultivation costs and labor requirements, and significantly reducing crop yields. These harmful species exhibit invasive growth patterns through resource and spatial competition, negatively impacting cultivated plants (Wani et al., 2021). In fact, they compete aggressively with crops for essential resources, leading to yield losses that can reach up to 90% (Ekwealor et al., 2019; Kaur et al., 2019). In addition, some species produce toxins that can poison livestock through consumption (Ekwealor et al., 2019). Furthermore, weeds thrive in diverse ecosystems such as pastures, forests, and ragelands (Wani et al., 2021), thereby disturbing ecological balance, reducing native biodiversity, and degrading the aesthetic quality of natural landscapes. Their widespread distribution makes them a major global concern for both environmental and economic sectors due to their harmful effects (Ekwealor et al., 2019). Globally, heavy weeds infestations pose serious threats to food security, public health, economic stability, and environmental sustainability (Fried et al., 2017; Pacanoski and Mehmeti, 2021). Therefore, efficient weed management is essential for maintaining agricultural productivity and sustainability (Verma et al., 2015; Aparna et al., 2024).

Among weed management strategies, chemical control is a highly effective method for weed mitigation, accounting for the majority of pesticide use worldwide (Alsaadawi et al., 2020). However, their excessive and indiscriminate use has raised serious concerns due to their ecological and health-related consequences, including soil degradation, environmental pollution, toxicity to non-target organisms, and the development of herbicide-resistant weeds (Fogliatto et al., 2020; Alsaadawi et al., 2020; Motmainna et al., 2021; Mesnage et al., 2021). Thus, reliance on synthetic herbicides presents a major challenge to achieving sustainable agriculture (Lykogianni et al., 2021).

Conventional methods are increasingly being replaced or complemented by novel approaches and eco-friendly alternatives (Alsaadawi et al., 2020; Aparna et al., 2024).

Integrated weed management, which combines preventive, cultural and biological control strategies, represents a cornerstone of sustainable weed control (Verma et al., 2015). Non chemicals approaches such as the use of cover crops, mowing, tillage, flaming, and hot-water treatments are gaining importance as viable substitutes (Fogliatto et al., 2020). Among these sustainable solutions, allelopathy stands out as a particularly promising and environmentally friendly method for sustainable weed suppression (Kostina-Bednarz et al., 2023; Khamare et al., 2022; Rithiga et al., 2024).

The term allelopathy, introduced by Hans Molisch in 1937 from the Greek words *allelon* (mutual) and *pathos* (harm), describes the biochemical interactions among plants (Kumari et al., 2017). According to the International Allelopathy Society, allelopathy refers to the release of bioactive secondary metabolites (allelochemicals) by plants or microorganisms into the environment (Shan et al., 2023). These allelochemicals can either inhibit or stimulate the growth and development of neighboring plants (Scavo et al., 2018; Macias et al., 2019). These secondary metabolites are synthesized naturally or under abiotic (drought, light and temperature) and biotic (pathogens and herbivory) stress (Singh et al., 2021; Shan et al., 2023). They comprise various chemical classes including phenols, flavonoids, terpenoids, alkaloids, and nitrogenous compounds (Mushtaq et al., 2020; Xu et al., 2023). They are released via multiple pathways such as volatilization, leaching, root exudation, and plant residue decomposition (Scavo et al., 2018; Dahiya et al., 2018). These metabolites interfere with key physiological processes such as photosynthesis, mineral uptake, protein synthesis, membrane integrity, and oxidative balance (Cheng and Cheng, 2015; Aziz et al., 2021). A frequent mode of action involves the overproduction of reactive oxygen species, resulting in oxidative stress and cellular damage in recipient plants (Staszek et al., 2021). The application of allelopatic residues or plant extracts is among the most viable and practical and eco-friendly methods for weed suppression (Alsaadawi et al., 2020). This can be achieved through cover cropping, crop rotation, mulching, and intercropping with allelopathic species (Mushtaq et al., 2020).

In this context, *Cleome arabica* L., *Diplotaxis harra* (Forssk.) Boiss, *Nerium oleander* L., and *Retama raetam* (Forssk.) Webb., four spontaneous plants collected from arid regions of Tunisia, were investigated for their allelopathic potential on the germination and seedling growth of two weeds *Setaria verticillata* L (bristly foxtail) and *Sisymbrium irio* L (London rocket) and two crops *Capsicum annuum* L (pepper) and *Solanum lycopersicum* L (tomato). Additionally, the bioactive composition of these plants, particularly their polyphenols, flavonoids, and tannins, was analyzed to elucidate the biochemical basis of their allelopathic activity alongside their antioxidant potential. The phytotoxicity results were supported by an *in silico* molecular docking study. Our findings highlight the promising use of these species as natural herbicides for sustainable and environmentally responsible weed management.

## 2 Materials and methods

### 2.1 Vegetal materials

The selected plants (*C. arabica* L., *D. harra* (Forssk.) Boiss*, N. oleander* L., and *Retama raetam* (Forssk.) Webb.) were collected at the vegetative stage from the region of Medenine (southern Tunisia) in 2024. The seeds of *S. verticillata* L. were collected from the same region and period, while the seeds of *S. irio* L. were collected from the region of Gabes (Tunisia) in the same year. Seeds of *C. annuum* L (pepper) and *S. lycopersicum* L (tomato) were purchased from a local market located in Mednine, Tunisia. The aerial parts of the four plants were harvested, rinsed with distilled water to remove any surface contaminants, and shade-dried in a ventilated dark chamber. Afterwards, the dried materials were ground using an electric grinder to obtain homogenous fine powders for extraction.

### 2.2 Preparation of extracts

Aqueous extracts of *C. arabica, D. harra*, *N. oleander* and *R. raetam*, were prepared by cold maceration in distilled water (10 g of plant powder in 100 mL of solvent) for 24 h with continuous agitation at 150 rpm using an incubator agitator (ORBITAL INCUBATOR SI50, Stuart). After filtration with filter paper, the extracts were lyophilized using a freeze dryer (ALPHA 1-4 LSC, Christ) to obtain dry powders. These were subsequently used for the concentration’s preparation, biochemical characterization, antioxidant assays, germination, and growth parameters.

### 2.3 Phytochemical screening

#### 2.3.1 Determination of total polyphenol content

Total polyphenol content in the aerial parts of the four plants was quantified using the modified Folin-Ciocalteu Reagent method as described by Shi et al. (2019). This colorimetric assay is based on the reduction of Folin-Ciocalteu Reagent by phenolic compounds under alkaline conditions, the intensity of which is proportional to polyphenol concentration. For analysis, 125 µL of each diluted extract was mixed with 500 µL of distilled water and 125 µL of Folin-Ciocalteu Reagent. After 3 min of vortex mixing (RX3, Velp Scientifica), 1250 µL of 7% sodium carbonate (Na_2_CO_3_) solution was added, and the final volume was adjusted to 3 mL with distilled water. Samples were incubated for 1 h in the dark at room temperature. Absorbance was measured at 760 nm using a microplate reader (VARIOSKAN FLASH, Thermo Fisher Scientific). All samples were analyzed in quadruplicate. A gallic acid (C_7_H_6_O_5_) standard curve (10, 50, 100, 150, and 200 μg/mL) was used for calibration and results were expressed as milligrams of Gallic acid equivalent (GAE) per Gram of dry weight (mg GAE/g DW).

#### 2.3.2 Determination of total flavonoid content

The total flavonoids content was assisted by the modified protocol based on Abdelwahab and Ashour (2018). The method is based on the formation of a flavonoid-aluminum chloride complex, which yields a chromophore measurable at 510 nm. Briefly, 250 µL of each diluted extract was mixed with 75 µL of 5% sodium nitrite (NaNO_2_) and incubated for 6 min at room temperature. Then, 150 µL of freshly prepared 10% aluminum chloride (AlCl_3_) was added, and incubated for 5 min. Finally, 500 µL of sodium hydroxide (NaOH) was added, and the total volume was adjusted to 2.5 mL with distilled water. After gentle mixing, absorbance was measured at 510 nm. All analyses were performed in quadruplicate. Catechin (C_15_H_14_O_6_) was used as the standard (10–400 μg/mL), and results were expressed as milligrams of catechin equivalents per Gram of dry weight (mg CE/g DW).

#### 2.3.3 Determination of condensed tannin content

Condensed tannin content was determined using a modified vanillin method adapted from Galgano et al. (2021). The assay is based on the depolymerization and transformation of condensed tannins into red-colored anthocyanidins in the presence of vanillin and concentrated sulfuric acid. 500 μL of each diluted extract was mixed with 3 mL of 4% vanillin (C_8_H_8_O_3_) in methanol and 1500 µL of sulfuric acid (H_2_SO_4_). The reaction mixture was incubation for 15 min at room temperature and absorbance were recorded at 500 nm. Catechin was used as a reference standard, and results were expressed as milligrams of catechin equivalents per Gram of dry weight (mg CE/g DW). All analyses were carried out in quadruplicate.

#### 2.3.4 LC-MS analysis

Liquid Chromatography (LC) analysis was conducted using a Shimadzu LC-20ADXR pump coupled with a SIL-20AXR autosampler maintained at 40 °C following the method of Ayaz et al. (2005). The injection volume was 5 μL, and separations were performed on a DiscoVery BIO Wide Pore C18 column (250 mm × 4 mm, 5 µm) at 75 °C. The separations were implemented at a flow rate of 0.4 mL/min. The mobile phase consisted of two eluents. Eluent A: methanol/water (5:95, v/v) containing 0.15% acetic acid and eluent B: acetonitrile/water (50:50, v/v) containing 0.15% acetic acid. The gradient program was used as: 0–14 min (10%–20% eluent B), 14–27 min (20% eluent B), 27–37 min (20%–55% eluent B), 37–45 min (55% eluent B), and 45–52 min (55%–100% eluent B). Mass spectrometry (MS) analysis was performed using a Shimadzu UFLC XR–2020 single-quadrupole mass spectrometer with an electrospray ionization interface (ESI) operating in negative ion mode. Selected ion monitoring (SIM) was operated under the following conditions: source temperature 450 °C, desolvation temperature 280 °C, capillary voltage 2.7 kV, cone voltage 35 V, nebulizing gas flow 1.5 L/min, and a drying gas flow 15 L/min. Phenolic compounds were identified by comparing their retention times and mass spectra with authentic standards.

#### 2.3.5 Antioxidant capacity

##### 2.3.5.1 Total antioxidant capacity

Total antioxidant capacity of was determined according to Phatak and Hendre (2014), with minor modifications. This method is based on the reduction of molybdenum (VI) (MoO_3_) to molybdenum (V) (MoO_2_^+^) by the tested extract, resulting in a green phosphate-Mo (V) complex under an acidic condition. Each diluted extract (100 µL) was added to 1 mL of acidic reagent and incubated at 90 °C for 90 min in the dark. After cooling, the absorbance was recorded at 695 nm. All samples were analyzed in quadruplicate. The total antioxidant capacity concentration was assisted using ascorbic acid standard curve (10, 50, 100, 150, 200 and 250 μg/mL) and expressed as milligrams of ascorbic acid equivalent (AAE) per Gram of dry weight (mg AAE/g DW).

##### 2.3.5.2 DPPH antiradical capacity

The DPPH (2,2-diphenyl-1-picrylhydrazyl) radical scavenging activity was determined following Abdelwahab and Ashour (2018), with modifications. The DPPH^•^ radical reacts with hydrogen-donating antioxidants, leading to a color change from deep violet to light yellow.

Each extract (1.5 mL, at concentrations of 50–500 μg/mL) was mixed with 1.5 mL of DPPH solution and incubated for 30 min in the dark. Absorbance was measured at 517 nm. All analyses were performed in quadruplicate.

The percentage of inhibition was calculated as follows:

$$Inhibition\;\left(\%\right)=\left[\frac{\left(C-E\right)}{C}\right]x\;100$$

where **C** is the control absorbance and **E** is the extract absorbance.

### 2.4 Germination test

Germination assays were performed to assess the allelopathic potential of the extracts, following the methods of Jovičić et al. (2019) and Bertolacci et al. (2018). In this experiment, seeds were treated with plant extracts under controlled laboratory conditions (Friedman, 2017).

Twenty sterilized seeds (2% ethanol, 10 min) of each test species (weed or crop) were placed on moistened filter paper (Whatman No.1) and treated with 2 mL of extract at concentrations of 0.05, 10, and 20 g/L. Distilled water served as the control, with four replicates per treatment. The dishes were then incubated in a germination chamber under controlled conditions (25 °C ± 2 °C, 16/8 h light/dark cycle) for 7 days. Daily monitoring was carried out to record germinated seeds (radicle length ≥2 mm), which were subsequently removed. The same parameters were applied for the four studied plants. The following germination parameters were then calculated, following Al-Ansari and Ksiksi (2016): Germination Rate (GR), Germination Index (GI), and Mean Germination Time (MGT).

$GR=\left(\frac{TS}{S}\right)\times 100$ , where TS = total germinated seeds and S = number of initial seeds

$GI=\sum \left(\frac{SDi}{Di}\right)$ , where SDi = germinated seeds on day i and Di = day i

$MGT=\sum \frac{\left(SDi\times Di\right)}{St}$ , where St = total seeds tested.

### 2.5 Growth test

Growth parameters of shoots and roots serve as a primary method to assess allelopathic effects in plants. They involve exposing seedlings to extracts from allelopathic plants, followed by measuring the shoot and root lengths (Rob and Noguchi, 2019).

Growth inhibition assays were performed according to Govea et al. (2020), with modifications. Germinated seeds (radicle ≥2 mm) were transferred to Petri dishes containing the same extract concentrations used in germination tests. Distilled water served as the control. Each dish contained 10 uniform seedlings, with four replicates per treatment for each plant species. Plates were maintained in the same germination conditions for 7 days, and irrigated daily with the extracts at different concentrations. Root and shoot lengths were measured, and growth inhibition or stimulation (G%) was calculated as follows (Andrianandrasana et al., 2020):

$$G\left(\%\right)=\left(\frac{\left(extract\;treated\;length-control\;length\right)}{control\;length}\right)\times 100$$

### 2.6 Molecular docking procedure

In order to predict the affinities and interaction modes between the selected molecules and the binding sites of the target enzymes, the AutoDock Vina software (Trott and Olson, 2010) was used to perform docking analyses of three major polyphenols (>100 ppm) identified during the phytochemical investigation. These compounds were docked into the active sites of two enzymes: glutathione S-transferase (GST; PDB: 1BX9) and 4-hydroxyphenylpyruvate dioxygenase (HPPD; PDB: 6J63). Structures of the selected molecules were designed and optimized utilizing 3D Viewer ACD software (http://www.filefacts.com/acd3d-viewer-freeware-info). The crystal structure of the GST and HPPD complexed with their co-crystallized inhibitors were retrieved from the Protein Data Bank (https://www.rcsb.org). The active site coordinates were determined through the positions of the co-crystallized inhibitors. Water molecules and co-crystallized ligands were eliminated. Subsequently, Kollman charges and polar hydrogens were added. Molecular docking for GST was conducted using a grid box centered on the enzyme’s 3D-structure at coordinates (16.980, 29.265, 21.156), with a grid size of 35 × 35 × 35 points. In contrast, docking simulations for HPPD were performed using a grid box centered at (29.532, −22.712, 4.898), also with a grid size of 35 × 35 × 35 points. For both enzymes, docking calculations were carried out with a grid spacing of 0.375 Å, an exhaustiveness parameter set to 8, and the number of binding modes fixed at 9. The docking protocol was validated by redocking the co-crystallized ligands into their respective binding sites under the described parameters. The resulting RMSD values were 1.5741 Å for GST and 0.9666 Å for HPPD, confirming the reliability of the docking procedure. Interaction profiles of the docked molecules within enzyme active sites were analyzed using DSV BIOVIA (2017).

### 2.7 Statistical analysis

All data were analyzed using SPSS software (v.11.5). One-way ANOVA was performed followed by Duncan’s multiple range test (p < 0.05). Results are expressed as mean ± standard error of quadruplicate measurements.

## 3 Results and discussion

### 3.1 Phytochemical screening

#### 3.1.1 Phenolic compounds screening

Allelopathy is a biochemical mechanism mediated by the release of phytotoxic metabolites that can influence the growth of neighboring plants. Among these, phenolic compounds represent a diverse class of allelochemicals known to inhibit key physiological processes such as germination, root elongation, and nutrient assimilation (Mushtaq and Fauconnier, 2024). Still, it is important to note that several metabolites are classified as pan-assay interfering compounds (PAINS), and so their biological activity should be interpreted with caution, especially when inferred from *in vitro* or *in silico* approaches. The secondary metabolites profile of the aqueous extracts of the four studied plants is presented in Table 1. Quantitative analysis revealed marked differences in the contents of total polyphenols, flavonoids, and tannins among the species. *Retama raetam* exhibited the highest concentrations of these bioactive compounds, with polyphenols 73.42 ± 0.32 mg GAE/g DW, flavonoids 76.59 ± 0.37 mg CE/g DW, and tannins 74.48 ± 0.31 mg CE/g DW, significantly exceeding those of *N. oleander*, with polyphenols 40.33 ± 0.54 mg GAE/g DW, flavonoids 68.80 ± 0.27 mg CE/g DW, and tannins 69.76 ± 0.53 mg CE/g DW) as well as *C. arabica* and *D. harra*. Our findings demonstrated a significantly higher polyphenols compounds contents in *R. raetam* compared to previous studies. For instance, the polyphenols content surpassed the one reported by Saada et al. (2018) (23.9 mg GAE/g). Similarly, its flavonoid content was also higher than the one reported by Rejab and Ksibi (2019) for *Retama retama* (5.04–5.76 mg CE/g DW) collected from the same region, obtained by maceration and Soxhlet methods, respectively. Its tannins content was importantly higher than the ones reported by Boussahel (2018) for *R. retama* aqueous extracts, collected from Algeria (3.22 mg CE/g DW). As a xerophytic shrub endemic to Mediterranean ecosystems, *R. raetam* displays remarkable allelopathic potentials and strong adaptations to arid conditions (Youssef et al., 2024). Its elevated polyphenol and tannin levels are closely associated with its allelopathic and antimicrobial activity, which has been shown to inhibit the growth of many weeds and phytopathogens (El-Darier et al., 2018; Soriano et al., 2022). These attributes, combined with its resilience in stressful environments, qualify *R. raetam* a valuable candidate for sustainable agroecology applications (Youssef et al., 2024).

**TABLE 1:** Phenolic compounds quantification of the aqueous extracts of *C. arabica*, *Diplotaxis harra*, *Nerium oleander* and *Retama raetam* collected from Tunisian arid regions. The letters (a, b, c, and d) indicate a significant difference at a threshold of 5%.

Secondary metabolites	Plants			
	*C*. *arabica*	*D. harra*	*N*. *oleander*	*R*. *raetam*
Polyphenols (mg GAE/g DW)	17.31 ± 0.15^c^	15.61 ± 0.30^c^	40.33 ± 0.54^b^	73.42 ± 0.32^a^
Flavonoids (mg CE/g DW)	38.09 ± 0.21^c^	30.01 ± 0.22^d^	68.80 ± 0.27^b^	76.59 ± 0.37^a^
Tannins (mg CE/g DW)	41.16 ± 0.50^c^	26.02 ± 0.25^d^	69.76 ± 0.53^b^	74.48 ± 0.31^a^

Similarly, *N. oleander* accumulates significant amounts of flavonoids and tannins, phytochemicals that play key roles in allelopathic interactions (Chaudhary et al., 2015; Sinha and Biswas, 2016). In addition, screening analyses have identified several bioactive compounds in *N. oleander* such as quercetin derivatives, which contribute to its inhibitory effects on seed germination and early seedling growth in neighboring plants (Assia et al., 2022). However, these metabolites are discussed as part of a complex phytochemical mixture, and their contribution to the observed effects is interpreted in conjunction with the experimental germination and growth results rather than as isolated pharmacologically active compounds.

#### 3.1.2 LC-MS profile

The LC-MS analysis disclosed considerable differences in the concentration and composition of phenolic composition among the four studied species. Table 2 reveals these differences, underlining the chemical diversity and allelopathic potentials of the studied plants.

**TABLE 2:** LC-MS anaylisis of *C. arabica*, *Diplotaxis harra*, *Nerium oleander* and *Retama raetam* collected from Tunisian arid regions.

Compound/Plant species	*C. arabica*	*D. harra*	*N. oleander*	*R. raetam*
Quinic acid	11.85	4.90	49.37	176.21
Chlorogenic acid	0.47	​	142.77	0.99
Rutin	​	​	203.44	4.38
Hyperoside	0.27	2.25	10.09	​
Quercetrin	0.86	8.62	2.61	5.01
Quercetin	​	1.75	1.82	​
Salviolinic acid	​	​	​	9.50
Naringenin	0.59	0.64	0.29	10.27
Naringin	24.08	​	3.44	​
Apegenin7-*O*-glucoside	​	0.023	​	​
Apegenin	0.14	​	​	13.17
Luteolin	2.45	​	​	68.02
Cirsiliol	0.39	0.53	0.12	0.17
Cirsilineol	0.50	5.52	​	​

Phenolic acids, widespread secondary metabolites in plant tissues, play an important role in allelopathy via numerous mechanisms. They are released into the soil through decomposition, root exudation, or leaching from living and decaying plant material. These acids affect plant growth by altering phytohormone activity, ion leakage, enzyme function, nutrient uptake, membrane permeability, and hydraulic conductivity (Li et al., 2002). Due to their structural diversity, rapid biodegradation, and limited environmental persistence, phenolic allelochemicals represent promising eco-friendly alternatives to synthetic herbicides (Mushtaq and Fauconnier, 2024). The allelopathic activity of phenolic acids depend not only on individual metabolites activity but also on synergistic interactions, their physical state and specific environmental concentrations (Li et al., 2002; Xie et al., 2014). Among the analyzed species, *R. raetam* showed the highest concentration of quinic acid (176.21 ppm) and was the only species containing measurable salviolinic acid. It also had the highest levels of luteolin (68.02 ppm), apigenin (13.17 ppm) and naringenin (10.27 ppm). *Nerium oleander* exhibited the highest concentration of rutin (203.44 ppm), chlorogenic acid (142.77 ppm), and hyperoside (10.09 ppm). Both *N. oleander* and *D. harra* contained comparable content of quercetin; however, *D. harra* displayed significant quantities of quercetrin (quercetin-3-*O*-rhamonosid) (8.62 ppm) and cirsilineol (5.52 ppm). Among all four species, *C. arabica* was distinguished by its high naringin content (24.08 ppm). Quinic acid and its derivatives are involved in multiple physiological processes, influencing seed germination and plant–pathogen interactions through both antioxidant and phytotoxic mechanisms (Huang et al., 2024; Gomes et al., 2020). Rutin, a well-known plant flavonoid, has been reported to inhibit seed germination and seedling growth in various plant species through multiple physiological and biochemical mechanisms, including the induction of oxidative stress, enhanced membrane lipid peroxidation, and alterations in leaf nutrient uptake (Hussain and Reigosa, 2014). Chlorogenic acid also modulates germination by suppressing radicle elongation in a dose-dependent manner, highlighting its regulatory role in early seed germination (Huang et al., 2024).

#### 3.1.3 Antioxidant capacity

Allelopathy involves plant–plant interactions mediated by chemical compounds that can induce oxidative stress in recipient species. Allelochemicals can stimulate the production of reactive oxygen species (ROS) and alter the cellular antioxidant defense systems, including hydroperoxidase, superoxide dismutase, and ascorbate-glutathione cycle enzymes. However, not all allelochemicals act as prooxidants. Some of them display antioxidant properties in a concentration-dependent way, eliciting variable physiological responses in target plants (Gniazdowska et al., 2015). The total antioxidant and antiradical capacities of the four selected species showed interspecific differences (Table 3). *Retama raetam* had the highest total antioxidant capacity (87.59 ± 0.85 mg AAE/g DW), followed by *N. oleander* (50.71 ± 0.63 mg AAE/g DW), *D. harra* (30.95 ± 0.80 mg AAE/g DW), and *C. arabica* (24.34 ± 0.78 mg AAE/g DW). Antiradical capacity followed a different pattern. *Nerium oleander* showed the lowest IC_50_ value (23.91 ± 0.02 μg/mL), indicating relatively higher scavenging activity, followed by *R. raetam* (79.62 ± 0.20 μg/mL). *Cleome arabica* and *D. harra* showed higher IC_50_ values exceeding 270 μg/mL. Statistical analysis confirmed significant interspecific variations (p < 0.05) for both capacities. These results are based on preliminary chemical assays and do not directly reflect biological activity. Djeddi et al. (2013) and Saada et al. (2018) attributed the antioxidant potential of *R. raetam* to its content and variety of phenolic compounds and other secondary metabolites. Phenolic compounds and flavonoids, present in *Retama* extracts, can act as free-radical scavengers (Benkhouili et al., 2024; Belmokhtar and Harche, 2014; Outaki et al., 2023). This antioxidant capacity may contribute to its allelopathic effect by affecting ROS levels in target species (Gniazdowska et al., 2014), probably influencing their growth and development (Aniya et al., 2022). Such mechanisms of *R. raetam*’s allelopathic effect may disrupt cellular processes in susceptible plants, with possible growth inhibition (Benkhouili et al., 2024; Outaki et al., 2023). Siham et al. (2014) reported antiradical capacity in various *N. oleander* extracts, with IC_50_ values ranging between 5 and 430 μg/mL. Studies on different plant parts of *N. oleander* (leaves, stems, and flowers) have described the presence of potent antioxidant compounds that contribute to radical scavenging and reducing capacities (Ali et al., 2010; Singhal and Gupta, 2012; Gayathri et al., 2012; Germi et al., 2013; Assia et al., 2022). The antioxidant capacity of this species may enhance its allelopathic potential by improving the effectiveness and stability of its allelochemicals, as well as influencing target plant responses (Bouabidi et al., 2025; Atay et al., 2018). The antioxidant defense mechanism of *N. oleander* contributes to its allelopathic behavior, possibly through effects on ROS dynamics in its environment (Kumar et al., 2016; 2017). Further studies should identify additional antioxidant substances in these species to clarify their role in allelopathy.

**TABLE 3:** The antioxidant capacity of *C. arabica*, *Diplotaxis harra*, *Nerium oleander* and *Retama raetam,* collected from Tunisian arid regions. The letters (a, b, c, and d) indicate a significant difference at a threshold of 5%.

Plant	Total antioxidant capacity (mg AAE/g DW)	Antiradical capacity (IC_50_ µg/mL)
*C. arabica*	24.34 ± 0.78^d^	271.30 ± 0.11^c^
*D. harra*	30.95 ± 0.80^c^	298.80 ± 0.06^d^
*N. oleander*	50.71 ± 0.63^b^	23.91 ± 0.02^a^
*R. raetam*	87.59 ± 0.85^a^	79.62 ± 0.20^b^

### 3.2 Germination test

**FIGURE 1:**
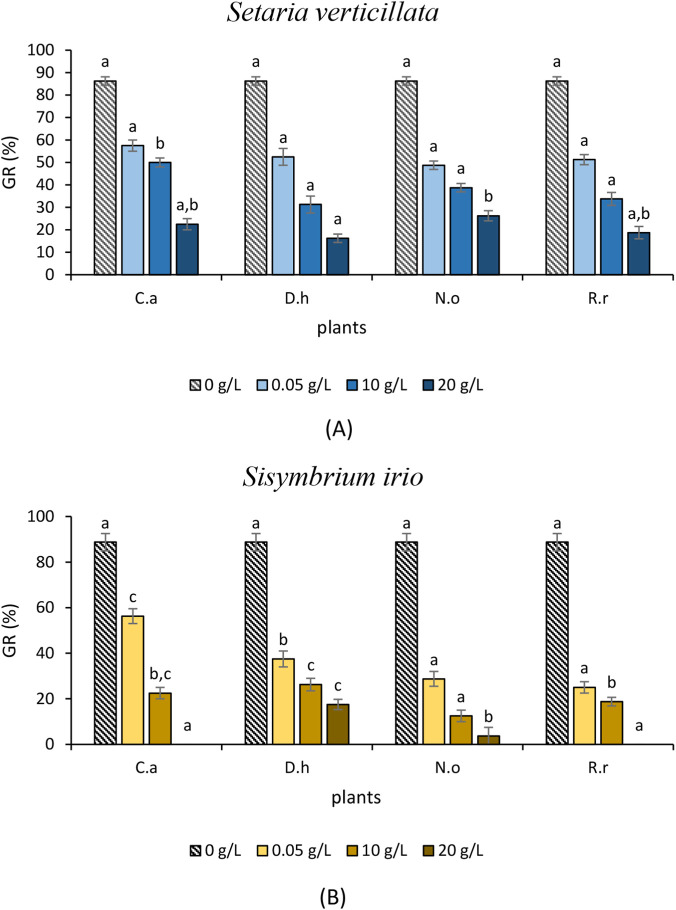
**(A)** Effect of the aqueous extracts of *Cleome arabica* (C.a), *Diplotaxis harra* (D.h), *Nerium oleander* (N.o), and *Retama raetam* (R.r) on *Setaria verticillata* Germination Rate (GR). The letters (a and b) indicate a significant difference at a threshold of 5%. **(B)** Effect of the aqueous extracts of *Cleome arabica* (C.a), *Diplotaxis harra* (D.h), *Nerium oleander* (N.o), and *Retama raetam* (R.r) on *Sisymbrium irio* Germination Rate (GR). The letters (a, b, and c) indicate a significant difference at a threshold of 5%.

**FIGURE 2:**
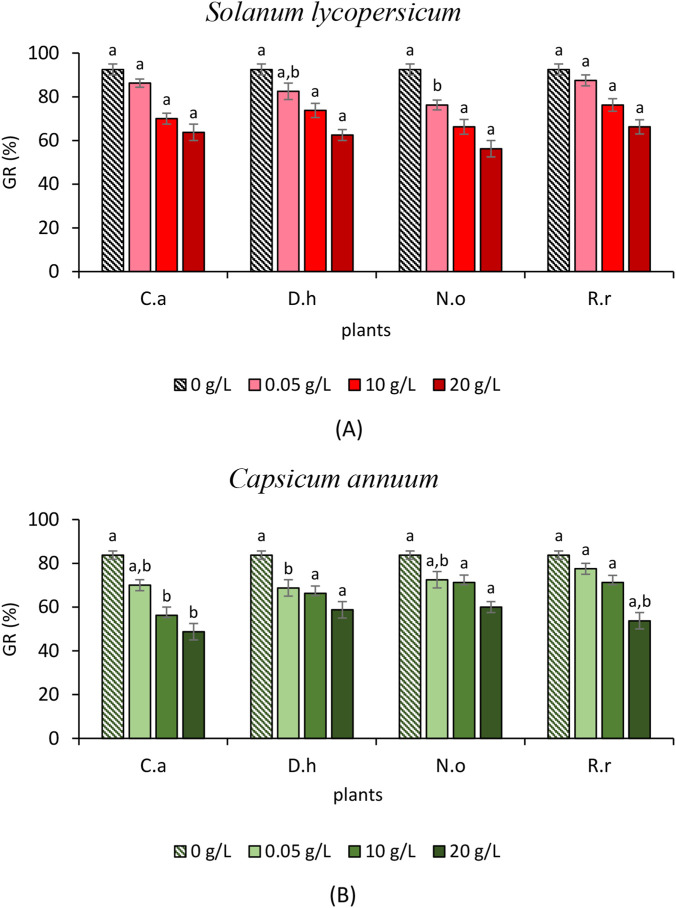
**(A)** Effect of the aqueous extracts of *Cleome arabica* (C.a), *Diplotaxis harra* (D.h), *Nerium oleander* (N.o), and *Retama raetam* (R.r) on *Solanum lycopersicum* Germination Rate (GR). The letters (a and b) indicate a significant difference at a threshold of 5%. **(B)** Effect of the aqueous extracts of *Cleome arabica* (C.a), *Diplotaxis harra* (D.h), *Nerium oleander* (N.o), and *Retama raetam* (R.r) on *Capsicum annuum* Germination Rate (GR). The letters (a and b) indicate a significant difference at a threshold of 5%.

To evaluate the allelopathic effects of the four plant extracts on seed germination of the tested weeds (*S. verticillata* L. and *S. irio* L.) and crops (*C. annuum* L. and *S. lycopersicum* L.), germination rate (GR), germination index (GI), and mean germination time (MGT) were determined. Figures 1, 2 present GR results, while Tables 4–7 summarize GI and MGT after exposure to three extract concentrations (5, 10, and 20 g/L).

**TABLE 4:** Effect of the aqueous extracts of *C. arabica*, *Diplotaxis harra*, *Nerium oleander* and *Retama raetam* on *S. verticillata* Germination Index (GI) and Mean Germination Time (MGT). The letters (a, b, and c) indicate a significant difference at a threshold of 5%.

​	GI				MGT			
​	Control	5 g/L	10 g/L	20 g/L	Control	5 g/L	10 g/L	20 g/L
*C*. *arabica*	4.66 ± 0.14^a^	3.04 ± 0.17^a^	2.66 ± 0.26^b^	1.26 ± 0.15^a^	2.91 ± 0.04^a^	3.73 ± 0.06^a^	3.95 ± 0.11^a^	4 ± 0.04^a^
*D*. *harra*		2.92 ± 0.21^a^	1.84 ± 0.22^a^	1 ± 0.11^a^		3.48 ± 0.06^b^	3.54 ± 0.23^a^	3.82 ± 0.11^a^
*N*. *oleander*		2.71 ± 0.18^a^	2.17 ± 0.24^a,b^	1.17 ± 0.57^a^		3.45 ± 0.05^b^	3.59 ± 0.11^a^	3.67 ± 0.28^a^
*R*. *raetam*		2.79 ± 0.32^a^	1.87 ± 0.31^a^	1.15 ± 0.23^a^		3.33 ± 0.04^c^	3.73 ± 0.32^a^	3.86 ± 0.12^a^

**TABLE 5:** Effect of the aqueous extracts of *C. arabica*, *Diplotaxis harra*, *Nerium oleander* and *Retama raetam* on *S*. *irio* Germination Index (GI) and Mean Germination Time (MGT). The letters (a, b, and c) indicate a significant difference at a threshold of 5%.

​	GI				MGT			
​	Control	5 g/L	10 g/L	20 g/L	Control	5 g/L	10 g/L	20 g/L
*C*. *arabica*	4.65 ± 0.22^a^	3.04 ± 0.26^a^	2.66 ± 0.16^c^	1.26^a^	2.91 ± 0.10^a^	3.73 ± 0.32^a^	3.95 ± 0.20^a^	4^a^
*D. harra*		2.92 ± 0.34^a^	1.84 ± 0.39^a^	0.95 ± 0.07^a^		3.48 ± 0.17^b^	3.54 ± 0.33^a^	3.79 ± 0.34^a^
*N*. *oleander*		2.71 ± 0.57^a^	2.17 ± 0.54^a,b^	1.03 ± 0.33^a^		3.45 ± 0.31^b^	3.58 ± 0.19^a^	3.72 ± 0.87^a^
*R*. *raetam*		2.79 ± 0.20^a^	1.87 ± 0.51^a^	1.15^a^		3.33 ± 0.23^c^	3.73 ± 0.10^a^	3.86^a^

**TABLE 6:** Effect of the aqueous extracts of *C. arabica*, *Diplotaxis harra*, *Nerium oleander* and *Retama raetam* on *S*. *lycopersicum* Germination Index (GI) and Mean Germination Time (MGT). The letters (a, b, and c) indicate a significant difference at a threshold of 5%.

​	GI				MGT			
​	Control	5 g/L	10 g/L	20 g/L	Control	5 g/L	10 g/L	20 g/L
*C*. *arabica*	6.57 ± 0.26^a^	6.15 ± 0.19^a^	4.91 ± 0.20^a^	4.47 ± 0.11^a^	3.47 ± 0.01^a^	3.34 ± 0.07^a^	3.30 ± 0.21^a^	3.25 ± 0.11^a^
*D*. *harra*		5.67 ± 0.26^a,b^	4.72 ± 0.24^a^	3.02 ± 0.51^a,b^		3.47 ± 0.06^b^	3.44 ± 0.20^a^	3.43 ± 0.14^a^
*N*.*oleander*		5.19 ± 0.50^c^	4.50 ± 0.36^a^	2.69 ± 0.34^a,b^		3.36 ± 0.14^a^	3.46 ± 0.08^a^	3.42 ± 0.10^a^
*R*.*raetam*		5.43 ± 0.66^a,b^	4.86 ± 0.21^a^	0.97 ± 0.05^c^		3.45 ± 0.12^a^	3.35 ± 0.13^a^	3.33 ± 0.05^a^

**TABLE 7:** Effect of the aqueous extracts of *C. arabica*, *Diplotaxis harra*, *Nerium oleander* and *Retama raetam* on *C*. *annuum* Germination Index (GI) and Mean Germination Time (MGT). The letters (a, b, and c) indicate a significant difference at a threshold of 5%.

​	GI				MGT			
​	Control	5 g/L	10 g/L	20 g/L	Control	5 g/L	10 g/L	20 g/L
*C*.*arabica*	4.07 ± 0.15^a^	3.45 ± 0.27^a^	2.69 ± 0.19^c^	2.41 ± 0.29^a^	3.20 ± 0.07^a^	3.0.2 ± 0.25^a^	2.93 ± 0.21^a^	2.86 ± 0.19^a^
*D*. *harra*		3.19 ± 0.18^a^	3.13 ± 0.20^a,b^	1.88 ± 0.34^a^		2.47 ± 0.14^c^	2.43 ± 0.19^c^	2.3 ± 0.10^a^
*N*. *oleander*		3.51 ± 0.29^a^	3.21 ± 0.22^a,b^	2 ± 0.10^a^		2.73 ± 0.23^b^	2.56 ± 0.11^a,b^	2.49 ± 0.12^a^
*R*.*raetam*		3.65 ± 0.32^a^	3.31 ± 0.31^a^	2.59 ± 0.15^a^		2.71 ± 0.08^b^	2.65 ± 0.19^a,b^	2.49 ± 0.20^a^

#### 3.2.1 Effects of the aqueous plant extracts on weeds germination

In *S. verticillata* (Figure 1A), all extracts at 5 g/L reduced germination by approximately half. Increasing concentrations intensified the inhibitory effects: *D. harra* caused the strongest inhibition, 68.75% and 83.75% at 10 and 20 g/L, respectively, followed by *R. raetam*, 66.25% and 81.25% at the same concentrations. *Cleome arabica* (77.50%) and *N. oleander* (71%) showed comparatively weaker while still significant reductions at 20 g/L. For *S. irio* (Figure 1B), *R. raetam* and *N. oleander* exhibited the strongest inhibitory effects at the lowest concentration (5 g/L), with GR being reduced by 75% and 71.25%, respectively. At 10 g/L, all extracts inhibited GR by more than 70%, with *N. oleander* showing the highest suppression (87.50%), followed by *R. raetam* (81.25%). At the highest concentration (20 g/L), *R. raetam* and *C. arabica* completely inhibited the germination, while *N. oleander* reduced it by 96.25%. These results demonstrate that allelochemicals can significantly inhibit germination even at low concentrations (5 g/L). Consistent with previous findings, phytotoxic metabolites can disrupt germination and metabolic functions at trace levels (Cheng et al., 2024; Anwar et al., 2021). The concentration-dependent response observed in *R. raetam* and *C. arabica*, which achieved total inhibition in *S. irio* at 20 g/L, aligns with the general pattern of allelopathy reported by Kanjevac et al. (2023), confirming a clear dose–response. Higher concentrations of allelochemicals increase bioavailability and thus phytotoxic intensity (Zhao et al., 2015). Interestingly, *N. oleander* displayed selective inhibition. It showed a strong effect on *S. irio* (96.25%), a dicotyledon speciecs, while a weaker effect on *S. verticillata* (71%), a monocotyledon speciecs. This species-specific sensitivity likely reflects the selectivity of allelochemical metabolites, suggesting that biochemical adaptation enforces allelopathic competitiveness in plant-plant interactions (Yuan et al., 2022). GI and MGT results (Tables 4, 5) support GR patterns. All extracts significantly reduced GI and MGT in *S. verticillata* and *S. irio* in a concentration-dependent manner. At 20 g/L, *D. harra* caused near-complete inhibition in *S. verticillata* (GI = 1 ± 0.11) and a similarly strong effect in *S. irio* (GI = 0.95), followed by *N. oleander* (GI = 1.03).

At the lowest tested concentration, all extracts decreased GI values compared to the untreated group. At 10 g/L, all tested extracts further decreased the GI, with *D. harra* and *R. raetam* showing the strongest inhibitory effects in *S. verticillata* and *S. irio.* At the maximum concentration (20 g/L), an important consistent decrease in GI values was observed particularly for *D. harra* in *S. verticillata* (GI = 1 ± 0.11), indicating nearly complete germination inhibition.

This observed reduction in GI corroborates the germination inhibition trends shown in Figure 1A, confirming that *D. harra* has the strongest phytotoxicity effect in *S. verticillata*. Similarly, this pattern intensified at 20 g/L, where *D. harra* and *N. oleander* exhibited substantial GI reduction of 0.95 and 1.03, respectively, in *S. irio. D. harra* extracts restrained the germination and seedling growth of other weeds arid regions plants (Hegazy et al., 1990). *D. harra*, rich in phenolics and other secondary metabolites (Falleh et al., 2013; Scavo et al., 2018), is known for its strong allelopathic effect, which likely underlies its inhibitory action observed in our study. Furthermore, all extract treatments consistently extended MGT compared to control groups. The MGT of *S. verticillata* and *S. irio* increased in a concentration-dependent manner, notably for *C. arabica* and *R. raetam,* at the highest concentration, indicating deleterious effects. However, *D. harra* and *N. oleander* showed moderate delays.

A lower GI and a higher MGT typically indicate an allelopathic inhibition potential, reflecting a reduced or delayed germination due to allelochemicals (Scrivanti and Anton, 2021; Oraon and Mondal, 2023). In contrast, an increased GI and a decreased MGT reflect a stimulatory allelopathic effect (Scrivanti and Anton, 2021). For instance, several studies demonstrated that aqueous extracts from many allelopathic plants can significantly inhibit germination rates and delay their onset in target plant species (Chen et al., 2014; Xie and Wang, 2024). The research conducted by Wu et al. (2011), Moosavi et al. (2011) confirmed that aqueous extracts from various plant parts can significantly inhibit GR and GI and delay MGT of target species, especially with increasing concentrations. Such inhibition may result from impaired respiration, protein synthesis inhibition, hormonal imbalance, and altered membrane permeability (Yang, 2005).

#### 3.2.2 Effects of the aqueous plant extracts on crops germination

Figure 2A presents the effect of the aqueous extracts on *S. lycopersicum* germination. At the lowest concentration, GR remained high (76.25%–87.50%) in *N. oleander* and *R*. *raetam*. At 10 g/L, GR slightly decreased but stayed above 66%, with *R. raetam* showing minimal effect. Even at 20 g/L, the inhibition was moderate. *Nerium oleander* showed the lowest GR (56.25%), while *R. raetam* maintained 66.25%. Figure 2B, presenting the effects in *C. annuum*, showed that the concentration of 5 g/L produced a negligible reduction compared to the control (83.75%), particularly in *R. raetam* (77.50%). The increase of the concentration to 10 and 20 g/L caused mild-to-moderate inhibition. For instance, *C. arabica* and *R. raetam* showed the lowest inhibitions at 20 g/L (48.75% and 53.75%, respectively). *Nerium oleander* (60%) and *D. harra* (58.75%) maintained germination rates above 50%, indicating a relatively high crop tolerance. GI data (Tables 6, 7) confirmed these trends. *Cleome arabica* and *D. harra* caused the weakest inhibition in *S. lycopersicum*, whereas *R. raetam* was the most suppressive (GI = 0.97 ± 5 at 20 g/L). Conversely, *R. raetam* had minimal effect on *C. annuum* (GI = 3.65 ± 0.32 at 5 g/L to 2.59 ± 0.15 at 20 g/L). MGT of *S. lycopersicum* remained unchanged across treatments, even at 20 g/L. In *C. annuum*, *D. harra* accelerated germination at all concentrations, suggesting a potential stimulatory effect at low doses. Previous studies reported that allelopathic compounds can either inhibit or stimulate crop germination depending on species sensitivity and concentration (Baek et al., 2017; Kamel and Hammad, 2015; Qasem, 2022). Studies on various crops, including tomato and pepper, have shown a dose-dependent response to phytotoxic extracts (Qasem, 2022). This variability may be linked to detoxification mechanisms allowing tolerant crops to neutralize allelochemicals and restore physiological balance (Bakhshayeshan-Agdam and Salehi-Lisar, 2020).

### 3.3 Growth test

**FIGURE 3:**
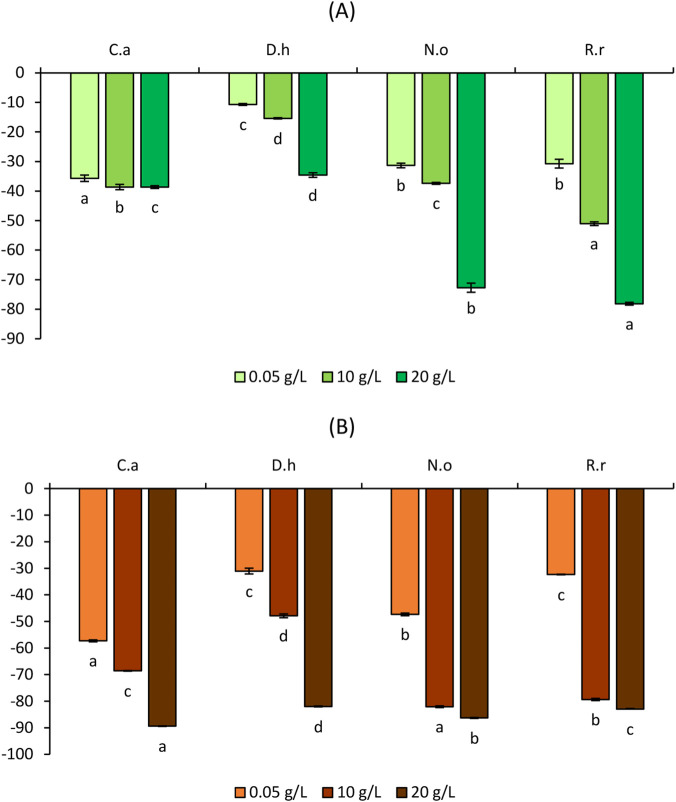
Effect of the aqueous extracts of *Cleome arabica* (C.a), *Diplotaxis harra* (D.h), *Nerium oleander* (N.o), and *Retama raetam* (R.r) on the growth of shoots **(A)** and roots **(B)** of *Setaria verticillata*. The letters (a, b,c, and d) indicate a significant difference at a threshold of 5%.

**FIGURE 4:**
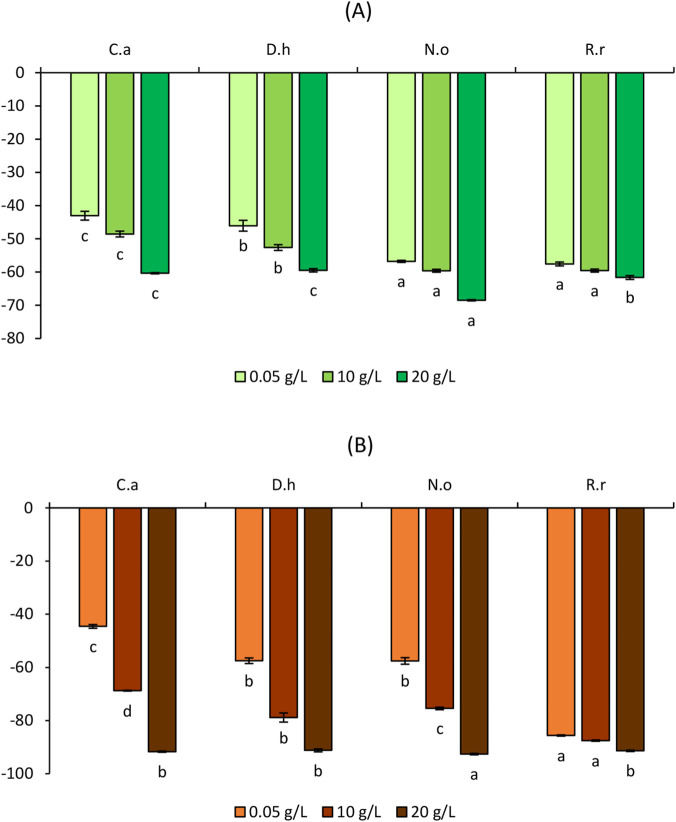
Effect of the aqueous extracts of *Cleome arabica* (C.a), *Diplotaxis harra* (D.h), *Nerium oleander* (N.o), and *Retama raetam* (R.r) on the growth of shoots **(A)** and roots **(B)** of *Sisymbrium irio*. The letters (a, b, and c) indicate a significant difference at a threshold of 5%.

**FIGURE 5:**
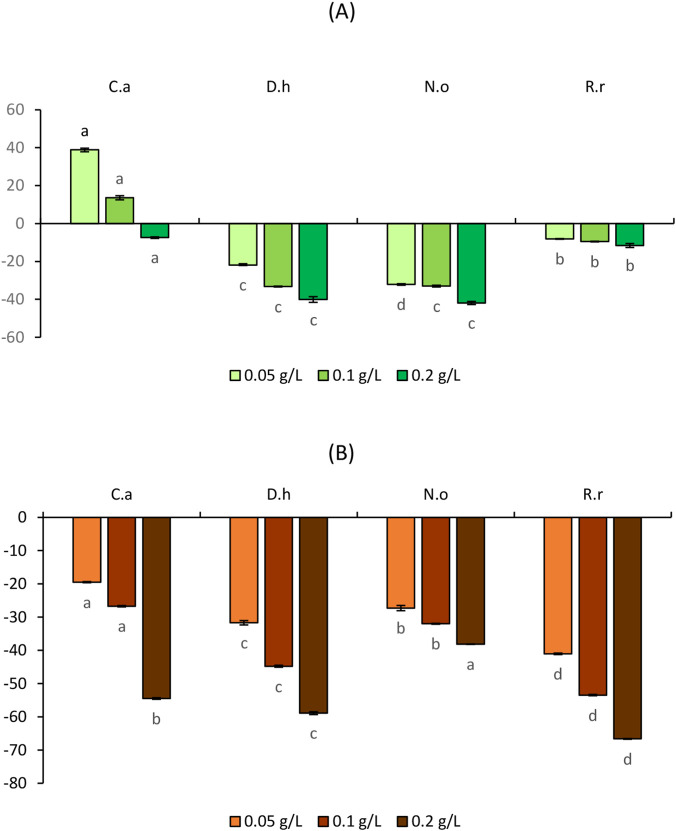
Effect of the aqueous extracts of *Cleome arabica* (C.a), *Diplotaxis harra* (D.h), *Nerium oleander* (N.o), and *Retama raetam* (R.r) on the growth of shoots **(A)** and roots **(B)** of *Solanum lycopersicum*. The letters (a, b, and c) indicate a significant difference at a threshold of 5%.

**FIGURE 6:**
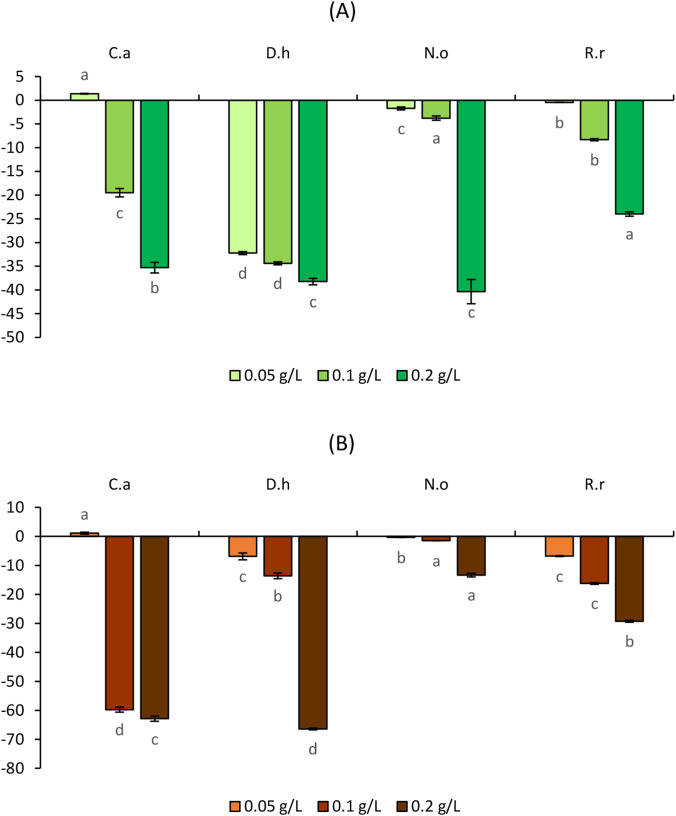
Effect of the aqueous extracts of *Cleome arabica* (C.a), *Diplotaxis harra* (D.h), *Nerium oleander* (N.o), and *Retama raetam* (R.r) on the growth of shoots **(A)** and roots **(B)** of *Capsicum annuum*. The letters (a, b, and c) indicate a significant difference at a threshold of 5%.

Figures 3–6 illustrate the effects of the four aqueous extracts on shoot and root growth of *S*. *verticillata, S*. *irio, S*. *lycopersicum,* and *C*. *annuum* after 7-day treatment at concentrations of 5, 10, and 20 g/L.

#### 3.3.1 Effects of the aqueous plant extracts on weeds growth

Across all concentrations, the shoots of *S. verticillata* experienced slight to moderate growth inhibition (Figure 3A). At 5 g/L, *D. harra* induced the weakest inhibition (−10.70% ± 0.31%), whereas *C. arabica* caused stronger suppression (−35.66% ± 1.09%) on the same weed. Increasing extract concentration intensified the growth inhibition. At 10 g/L, *R. raetam* showed the most pronounced effect (−51.02% ± 0.62%), while at 20 g/L, *R. raetam* (−78.15% ± 0.46%) and *N. oleander* (−72.72% ± 1.55%) caused severe shoot suppression. Roots of *S. verticillata* demonstrated higher sensitivity to allelochemicals than shoots (Figure 3B). At 5 g/L, *C. arabica* caused the highest inhibition (−57.30% ± 0.36%). Root suppression increased with concentration. At 10 g/L, *N. oleander*, *R. raetam*, and *C. arabica* exhibited shoot growth inhibition rates of −82.11% ± 0.33%, −79.35% ± 0.40%, and −68.58% ± 0.11%, respectively. At 20 g/L, growth inhibition exceeded 80% in all extracts and reached −89.42% ± 0.11% for *C. arabica*. In *S*. *irio,* shoot growth decreased progressively in all species (Figure 4A). At the minimal concentration, the inhibition ranged from −43.02% ± 1.33% (*C. Arabica*) to −57.56 %± 0.58% (*R. Raetam*). At 10 g/L, *N. oleander* (−59.59% ± 0.44%) and *R. raetam* (−59.55% ± 0.46%) exhibited similar strong effects. At the maximum concentration, inhibition exceeded 60% in all species, with *N. oleander* (−68.49% ± 0.19%) and *R. raetam* (−61.63% ± 0.58%), remaining the most potent inhibitors. Root systems were particularly sensitive to allelochemical metabolites, revealing near-complete growth suppression at the highest concentration (Figure 4B). Notably, *R. raetam* showed an inhibition of −85.61% ± 0.23% at 5 g/L and reaching −87.53% ± 0.25% at 10 g/L. *D. harra* (78.85% ± 1.71%) and *N. oleander* (−75.43% ± 0.47%) followed closely. At 20 g/L, the inhibition exceeded 90% for all species, with *N. oleander* reaching −92.61% ± 0.27%, indicating a critical toxicity threshold for root growth. The inhibitory effects of allelochemicals on weed growth include the suppression of essential physiological processes like seed germination, root elongation, and shoot development (Mali et al., 2021; Rasheed, 2020; Boukhili et al., 2022). Belz et al. (2022) and Moraes et al. (2023) confirmed that secondary metabolites released by plants inhibit the growth of neighboring species in a concentration-dependent and often species-specific manner. Both weed species in this study exhibited dose-dependent inhibition, with roots consistently more affected than shoots. This agrees with previous findings showing that root length is a highly sensitive biomarker of phytotoxic stress, as roots are directly exposed to allelochemicals in the soil (Zhou et al., 2021; Qu et al., 2021; Bakacsy et al., 2024). The stronger inhibition of root growth may result from disrupted auxin homeostasis, oxidative stress generation, and alterations in root tip ultrastructure (Šoln and Koce, 2021; Kumar et al., 2024; Choudhary et al., 2023; Šoln et al., 2022).

#### 3.3.2 Effects of the aqueous plant extracts on crops growth

The four plant extracts demonstrated distinct allelopathic effects in *S. lycopersicum* (Figure 5) and *C*. *annuum* (Figure 6). Overall, growth inhibition in crops was significantly milder than the ones observed in weed species. Furthermore, *C. arabica* stimulated shoot growth, particularly in *S. lycopersicum*. As shown in Figure 5A, shoot elongation was remarkably stimulated by it especially at the lowest dose (5 g/L), with a 38.93% ± 0.81% increase compared to the control. A moderate stimulation was also observed at 10 g/L (13.63% ± 1.09%). *Retama raetam* showed the weakest inhibitory effect across all concentrations, with reductions of only −8.11% ± 0.17%, −9.50% ± 0.17%, and −11.59% ± 1.04% at 5, 10, and 20 g/L, respectively. Both *D. harra* and *N. oleander* caused moderate effects, not exceeding 45% inhibition even at the highest concentration. Root growth (Figure 5B) was generally more sensitive, particularly to *R. raetam*, which showed notable inhibition at all doses. Conversely, *C. arabica* exhibited the weakest inhibition at 5 g/L (−19.47% ± 0.17%) and 10 g/L (−26.72% ± 0.20%). *Nerium oleander* also showed relatively mild inhibition (−38.14% ± 0.07%) at the highest concentration compared with the other species. *C. annuum* exhibited even greater tolerance in shoots compared to *S. lycopersicum*. *Cleome arabica* slightly stimulated shoot growth (1.38% ± 0.07%), while *R. raetam* demonstrated the weakest inhibition effects across all concentrations, with −0.46%, −8.32% ± 0.22%, and −23.98% ± 0.45% inhibition percentages with the increase of the concentrations (Figure 6A). Root growth (Figure 6B) was more affected than shoots, with *C. arabica* (−62.89% ± 0.89%) and *D. harra* (−66.44% ± 0.30%) showing the strongest inhibition at 20 g/L. In contrast, *N. oleander* and *R. raetam* exhibited the weakest inhibitory effects, never exceeding −30% inhibition. Notably, *N. oleander* showed the most promising tolerance profile, with minimal root inhibition of −0.31% ± 0.06%, −13.58% ± 0.06%, and −13.33% ± 0.63% at 5 g/L, 10 g/L, and 20 g/L, respectively.

Our findings are consistent with the principle of herbicide-induced hormesis, where low doses of phytotoxins compounds can stimulate plant growth by 10%–25% (Belz et al., 2011). Similarly, Xu et al. (2023) reported that low concentrations of potentially toxic compounds can trigger hormetic effects, enhancing photosynthetic activity and chlorophyll synthesis in tomato plants, ultimately promoting growth.

### 3.4 Molecular docking analysis

**FIGURE 7:**
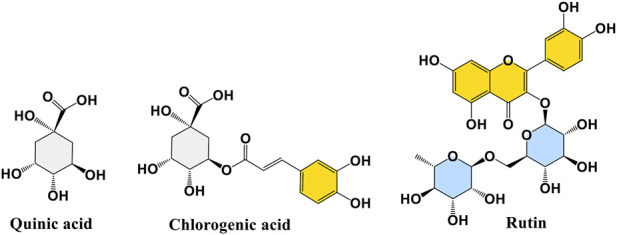
Molecular structures of quinic acid, chlorogenic acid and rutin.

Given the significant phytotoxic effects exhibited by *N. oleander* and *R. raetam* extracts, it becomes essential to investigate how their main constituents exert their activity. Molecular docking serves as an essential computational tool to elucidate how a bioactive compound may interact within the active site of a target enzyme. This *in silico* approach provides detailed insights into binding conformations and affinities, thereby enhancing the mechanistic understanding and corroborating data from *in vitro* and *in vivo* assays. Therefore, in this section of the study, the structures of quinic acid, chlorogenic acid and rutin (Figure 7) were docked within enzyme active sites of glutathione S-transferase (GST; PDB: 1BX9) and 4- hydroxyphenylpyruvate dioxygenase (HPPD; PDB: 6J63) to explore their potential interactions and affinities. Glutathione S-transferase (GST) was selected as a molecular target due to its pivotal role in plant detoxification systems and oxidative stress regulation. By catalyzing the conjugation of glutathione with toxic electrophilic compounds, GST contributes to the neutralization and removal of xenobiotics and reactive oxygen species.

**FIGURE 8:**
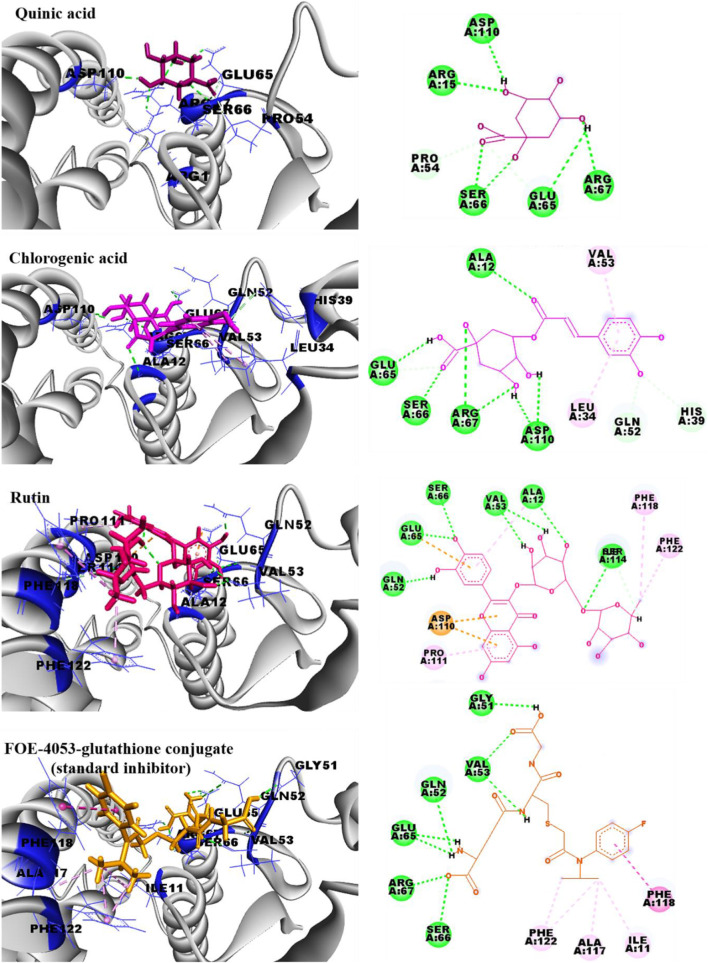
2D and 3D representations of the binding profiles of quinic acid, chlorogenic acid, rutin and FOE-4053-glutathione conjugate (GST standard inhibitor) with glutathione S-transferase enzyme (PDB: 1BX9).

**FIGURE 9:**
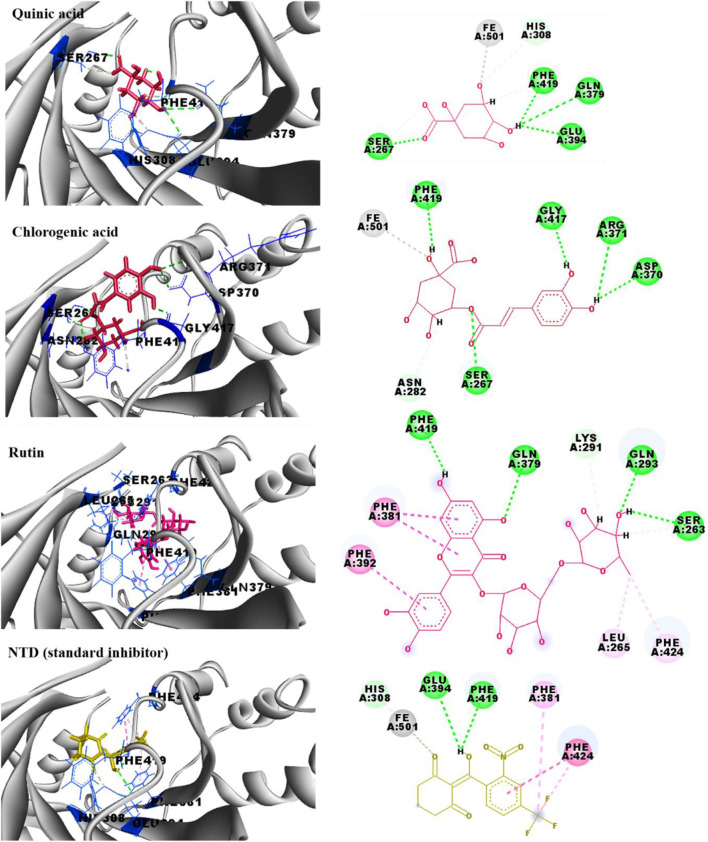
2D and 3D representations of the binding modes of quinic acid, chlorogenic acid, rutin and NTD (HPPD co-crystallized inhibitor) with 4-hydroxyphenylpyruvate dioxygenase enzyme (PDB: 6J63).

Inhibition of this enzyme can compromise cellular redox homeostasis, leading to oxidative damage that negatively affects seed germination and early plant development. In parallel, 4- hydroxyphenylpyruvate dioxygenase (HPPD) was chosen due to its essential function in the tyrosine degradation pathway, which is directly linked to the biosynthesis of plastoquinone and tocopherols. These metabolites are crucial for carotenoid production and protection of chlorophyll against photooxidative damage. Inhibition of HPPD results in carotenoid depletion, chlorophyll degradation, and disruption of photosynthetic processes, ultimately leading to growth inhibition and bleaching symptoms. Thus, targeting GST and HPPD provides a mechanistic framework that plausibly explains the phytotoxic effects observed in the germination and growth assays, thereby establishing a direct link between the molecular docking results and the experimental findings. Moreover, Glutathione S-transferase (GST) is pivotal in cellular detoxification processes, primarily catalyzing the conjugation of toxic electrophiles and oxidative stress byproducts with glutathione, thereby facilitating their elimination. Additionally, GST functions in hormone transport and modulation of cellular signaling pathways (Gullner et al., 2018). Conversely, 4-hydroxyphenylpyruvate dioxygenase (HPPD) is a critical enzyme involved in the catabolism of tyrosine, converting 4- hydroxyphenylpyruvate to homogentisate. Disruption of tyrosine degradation in plants results in severe physiological disturbances, including growth inhibition from tyrosine accumulation, oxidative stress due to diminished tocopherol (vitamin E) synthesis, and chlorophyll degradation stemming from a deficiency in carotenoids that normally protect chlorophyll molecules from photooxidative damage (Moran, 2005). Therefore, inhibition of these enzymes is a strategy used in some herbicides (Prade et al., 1998; Pan et al., 2019; Lin et al., 2019). From the *in silico* docking results, the binding energies of chlorogenic acid (−7.1 kcal/mol) and rutin (−7.8 kcal/mol) are better than that of the co-crystallized inhibitor (FOE-4053-glutathione conjugate; −6.7 kcal/mol), which will allow them to bind favorably to the active pocket of GST enzyme. The interaction profiles of the main identified polyphenols, all with that of the standard inhibitor of GST (FOE-4053-glutathione conjugate), are presented in Figure 8. Quinic acid (−4.9 kcal/mol) exhibited the weakest affinity for GST, while it carried out a good binding profile, displaying many similarities to that of the co-crystallized GST inhibitor. Quinic acid is involved in six conventional hydrogen bonds with Arg15, Pro54, Glu65, Ser66, Arg67 and Asp110 amino acids and two carbon hydrogen bonds with Pro54 and Glu65. Chlorogenic acid formed seven hydrogen bonds with Ala12, Glu65, Ser66, Arg67 and Asp110 residues, alongside two π-alkyl bonds with Leu34 and Val53, and two carbon hydrogen bonds with His39 and Gln52. The interaction profile of rutin is characterized by the presence of seven hydrogen bonds with the amino acids Ala12,Gln52, Val53, Glu65, Ser66 and Ser114, alongside three π-anion bonds with Glu65 and Asp110, four π-alkyl bonds with Ala12, Pro111, Phe118, and Phe122, and a carbon hydrogen bond with Ser114 residue. The co-crystallized GST inhibitor (FOE-4053-glutathione conjugate) formed eight conventional hydrogen bonds with Gly51, Gln52, Val53, Glu65, Ser66 and Arg67, two π-alkyl bonds with Phe122 residue, two alkyl ones with Ile11 and Ala117, and a π-π stacked bond with Phe118. From the *in silico* docking results within the binding site of the 4-hydroxyphenylpyruvate dioxygenase (HPPD; PDB: 6J63), the binding energies of chlorogenic acid (−8.0 kcal/mol) and rutin (−8.4 kcal/mol) are comparable to that of the co-crystallized inhibitor (NTD; −9.3 kcal/mol), which will allow them good affinities for HPPD enzyme. The interaction profiles of quinic acid, chlorogenic acid and rutin, all with that of the NTD (HPPD co-crystallized inhibitor), are presented in Figure 9. The binding profiles of the three docked polyphenols (quinic acid, chlorogenic acid and rutin) show many similarities with that of the co-crystallized HPPD inhibitor. The HPPD inhibitor (NTD; −9.3 kcal/mol) forms two conventional hydrogen bonds with Glu394 and Phe419, a metal-acceptor bond with the iron ion, a π-π stacked bond with Phe424, two π-alkyl bonds with Phe381 and Phe424 residues, and a carbon hydrogen bond with His308. On the other hand, quinic acid (-5.6 kcal/mol) is involved in four conventional hydrogen bonds with Ser267, Gln379, Glu394 and Phe419 residues, a metal-acceptor bond with the iron ion and three carbon hydrogen bonds with Ser267, His308 and Phe419 amino acids. Chlorogenic acid (−8.0 kcal/mol) binding mode is described by five hydrogen bonds with the Ser267, Asp370, Arg371, Gly417 and Phe419 amino acids, a metal-acceptor bond with the iron ion, and two carbon hydrogen bonds with Ser267, Asn282 residues. Rutin (−8.4 kcal/mol) is implicated in four hydrogen bonds with the Ser263, Gln293, Gln379 and Phe419 amino acids, three π-π stacked bonds with Phe381 and Phe392, a π-alkyl bond with Phe424, an alkyl bond with Leu265 and two carbon hydrogen bonds with Ser263 and Lys291 residues. The higher affinities and interaction profiles of the main polyphenols, identified in extracts of *N. oleander* and *R. raetam*, within the active sites of the target enzymes, predict a significant power to inhibit the targeted enzymes, which supports the significant phytotoxic results. Although molecular docking provides valuable insights into the binding modes and predicted affinities of ligands within enzymatic active sites, it remains an *in silico* approach primarily intended for preliminary and supportive predictions. Accordingly, the docking results presented herein should be considered as supplementary theoretical evidence and interpreted with caution, as they require further experimental validation to confirm their biological relevance.

## 4 Conclusion

This study highlights the significant allelopathic potential of *R. raetam* and *N. oleander* as promising eco-friendly candidates for sustainable weed management. Both species effectively inhibited weed germination and growth, while exerting minimal effects on tomato and pepper. Biochemical analyses revealed high levels of polyphenols, flavonoids, and tannins, along with distinctive LC–MS profiles, which that correlates with their allelopathic activity and antioxidant properties. In particular, *R. raetam* and *N. oleander* displayed remarkable total antioxidant and radical-scavenging capacities. The inhibition of weed germination and growth increased proportionally with extract concentration, indicating a dose-dependent response. In contrast, crop seeds-maintained germination rates above 50% even at 20 g/L, confirming their relative tolerance and the selectivity of these plant extracts. Growth bioassays further supported these findings, showing that root development was more sensitive than shoots, which exhibited only moderate inhibition. To support the phytotoxicity findings, a molecular docking study was conducted to predict the binding affinities and interaction profiles of the selected compounds, thereby providing computational insight into and support the experimental results. Overall, these results highlight *R. raetam* and *N. oleander* as sustainable alternatives to synthetic herbicides, contributing to the achievement of global sustainable agriculture goals. Future research should focus on large-scale field validation and the isolation of key bioactive compounds to optimize their efficiency and integration into environmentally friendly weed management strategies.

## Data Availability

The raw data supporting the conclusions of this article will be made available by the authors, without undue reservation.
